# The chromatin source-sink hypothesis: a shared mode of chromatin-mediated regulations

**DOI:** 10.1242/dev.201989

**Published:** 2023-11-03

**Authors:** Patrick J. Murphy, Frédéric Berger

**Affiliations:** ^1^ University of Rochester, Department of Biomedical Genetics and Department of Biology, 601 Elmwood Ave., Rochester NY 14620, USA; ^2^ Gregor Mendel Institute, Austrian Academy of Sciences, Vienna BioCenter; Dr. Bohr-Gasse 3, 1030 Vienna, Austria

**Keywords:** Chromatin, Epigenetics, Evolution, Transposons, Zygotic activation

## Abstract

We propose that several chromatin-mediated regulatory processes are dominated by source-sink relationships in which factors operate as ‘sources’ to produce or provide a resource and compete with each other to occupy separate ‘sinks’. In this model, large portions of genomic DNA operate as ‘sinks’, which are filled by ‘sources’, such as available histone variants, covalent modifications to histones, the readers of these modifications and non-coding RNAs. Competing occupation for the sinks by different sources leads to distinct states of genomic equilibrium in differentiated cells. During dynamic developmental events, such as sexual reproduction, we propose that dramatic and rapid reconfiguration of source-sink relationships modifies chromatin states. We envision that re-routing of sources could occur by altering the dimensions of the sink, by reconfiguration of existing sink occupation or by varying the size of the source, providing a central mechanism to explain a plethora of epigenetic phenomena, which contribute to phenotypic variegation, zygotic genome activation and nucleolar dominance.

## Teaser

**Summary:** A model in which cellular programming dynamics is governed by source-sink relationships, such that chromatin associated factors (‘sources’) compete to occupy regions of genomic DNA (‘sinks’).

## Introduction

All living organisms package their DNA with chromatin proteins. Chromatin proteins protect and compact the DNA (Ferrand et al., 2021; Hauer and Gasser, 2017; Zhou et al., 2019), facilitate the transmission of the genetic information to daughter cells (Stewart-Morgan et al., 2020), control the expression of the genetic information (Talbert and Henikoff, 2021; Teves et al., 2014) and likely participate in the evolution of genomes (Drinnenberg et al., 2019). Long before the identification of its molecular nature, it was recognized that chromatin exists in two main states: the densely stained heterochromatin contrasting with euchromatin (Heitz, 1928). Studies performed in mouse found heterochromatin to be located at repetitive regions (Jones, 1970; Pardue and Gall, 1970) and studies of *Drosophila* demonstrated that heterochromatin occurs at some inactive genes (Hartmann-Goldstein, 1966; Muller, 1930). Observations of *Drosophila* salivary glands revealed that genomic regions could switch between states of euchromatin and heterochromatin (facultative heterochromatin), whereas others remain continuously condensed (constitutive heterochromatin).

It was later discovered that euchromatic genes located near heterochromatin, often fail to be expressed in a measurable percentage of the cells, resulting in a mosaic pattern. This type of position effect has been termed variegation (reviewed by Lewis, 1950). Variegation applies to different traits, including the eye color in *Drosophila*, where differing degrees of expression arise through heterochromatin spreading outward from neighboring genomic loci into an eye color governing gene. It was also noted that the extent of variegation depends on the ratio of heterochromatin to euchromatin in the nucleus. The loss of the largely heterochromatic Y chromosome leads to the increase of a variegation in the X0 male compared with the XY male (Schultz, 1936). Likewise, the deficiency in a proportion of autosomal heterochromatin leads to increased variegation (Morgan et al., 1940).

In his seminal paper (Zuckerkandl, 1974), Emile Zuckerkandl proposed a model to explain how the distant action of large heterochromatic regions (male sex chromosomes) relieves the resident inhibition exerted by local insertions of heterochromatin on neighboring euchromatic loci. According to the model, histones of heterochromatin bind a molecule, X, that stimulates the conversion of neighbor euchromatin into heterochromatin with the consequent inhibition of transcription. If the total amount of heterochromatin increases in the cell, the effect of X on heterochromatin is diluted, resulting in lower impact of variegation. Zuckerkandl proposed that a ‘source-sink relationship’ governed the control of variegation, whereby regions of heterochromatin act as large sinks for chromatin-modifying factors acting as the source in this model. Based on current knowledge and recent observations, we propose a model that extends Zuckerkandl's ideas and is relevant of a broad spectrum of epigenetic regulations affecting a variety of developmental and physiological phenomenon. We also propose that applying the source-sink concept to development unites the molecular and Waddington's definitions of epigenetics, and envisage the role of epigenetic source-sink in evolution.

## The chromatin source-sink hypothesis

**Fig. 1.**
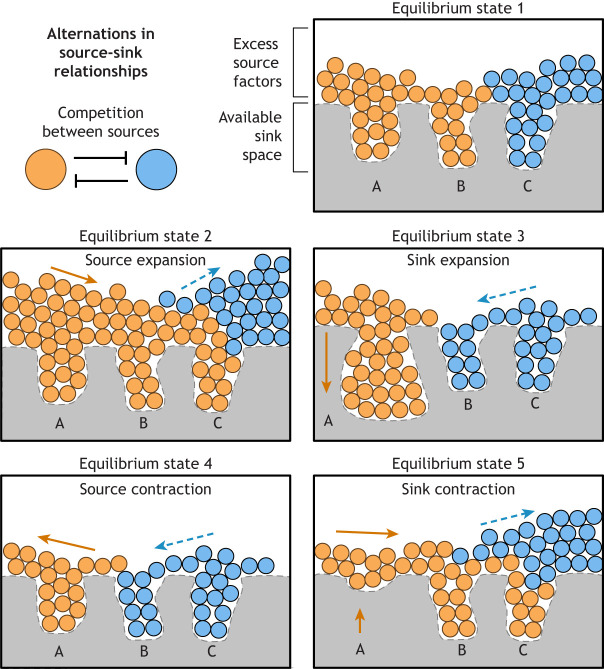
**Source-sink dynamics establish distinct epigenetic equilibriums.** Source factors are depicted as blue or orange marbles. These factors compete for residence within separate sinks, labeled as A, B or C. Five equilibrium states are depicted, each caused by altered source-sink relationships. Arrows indicate direct (orange) and indirect (dashed blue) impacts of source-sink dynamics. For equilibrium state 1, the orange source occupies sinks A and B, while the blue source occupies sink C. Expanding the orange source in equilibrium state 2, causes it to occupy sinks A, B and C, at the expense of the blue source. When sink A expansion occurs, in equilibrium state 3, the orange source resides completely within sink A, allowing the blue source to occupy sinks B and C. Likewise, when the orange source abundance decreases, leading to source contraction in equilibrium state 4, the blue source is able to occupy sinks B and C. Finally, when sink A contraction occurs (equilibrium state 5), the orange source is able to out-compete the blue source for sinks B and C.

Globally, chromatin is an assembly of factors (proteins and non-coding RNAs) that occupy discrete parts of genomic DNA. Competition between these factors occurs in a manner dependent on DNA affinity, chromatin turnover and overall factor abundance. Hence, chromatin dynamics revolves around sources of free factors, which flow into a restricted number of DNA sinks (Fig. 1). Here, we discuss the regulatory potentials of the interplay between the availability of sources and sinks.

Chromatin results from multiple interactions between its components. The basic unit of eukaryotic chromatin is the nucleosome comprising two copies of each core histone: two heterodimers of H2A-H2B and one tetramer of H3 and H4. Nucleosomes prevent access of transcription factors to DNA, and nucleosome remodelers are ATPases able to weaken the histone-DNA interaction and move nucleosomes to modulate access to transcription factor-binding elements (Clapier et al., 2017). Nucleosome properties are regulated by variants of the core histones. The deposition machinery specific to each histone variant controls the occupation of DNA (the sink) depending on the availability of sources: histone variants (Loppin and Berger, 2020; Martire and Banaszynski, 2020; Ray-Gallet and Almouzni, 2021; Talbert and Henikoff, 2021). The N- and C-terminal tails of histones that protrude from the nucleosome also act as sinks for post-translational modifications (PTMs) that are deposited or erased by (source) enzymes collectively referred to as ‘writers’ and ‘erasers’, respectively. Again at the next level of regulation, (PTMs) themselves can be viewed as sinks that are bound by ‘reader’ proteins (sources) that ‘translate’ the PTM code into a given function (Millán-Zambrano et al., 2022). As a concrete example: in fission yeast, the PTM trimethylation of lysine residue 9 of histone H3 (H3K9me3) is deposited by the methyltransferase Clr4, erased by the demethylase EpeI (Ragunathan et al., 2015; Trewick et al., 2007) and bound by Swi6 (the ortholog of heterochromatin protein 1) (Ekwall et al., 1995). Together, these proteins regulate the maintenance of heterochromatin that represses transcription (Grewal, 2023). In many eukaryotes, the deposition of PTMs and histone variants is guided or repelled by DNA methylation (Deleris et al., 2012; Du et al., 2014; Law and Jacobsen, 2010; Mathieu et al., 2005; Wollmann et al., 2017; Zilberman et al., 2008). Specific methyltransferases deposit 5-methylcytosine (5mC) at CpG sites, which is associated with the transcriptionally repressed status of transposons (Schmitz et al., 2019). The number of CpG sites defines the size of the sink that can be modified by the methyltransferases (viewed as the source), and the pattern obtained regulates binding of transcription factors and factors that control chromatin accessibility (Mattei et al., 2022).

Although chromatin is not separated by internal membrane compartments, it appears segregated into subdomains that might result from events comparable with phase separation. Various forms of organization have the potential to segment the chromatin into specialized sinks insulated from each other. For example, the nucleolus comprises the repetitive regions encoding ribosomal RNAs (rRNAs) (Picart-Picolo et al., 2019). Heterochromatin and euchromatin are further subdivided into specific combinations of PTMs and histone variants known as chromatin states, which associate with specific genomic regions and tend to localize at different areas of the nucleus (Jamge et al., 2023; van der Velde et al., 2021). One subtype of heterochromatin, for example, is more condensed and tends to occupy outer nuclear regions, at least partly via interaction with lamins located beneath the nuclear envelope (Manzo et al., 2022). Chromatin organization is also regulated in space and during the cell cycle by chromatin structural proteins (Di Stefano and Cavalli, 2022; Rowley and Corces, 2018). In interphase, chromatin loops produced by the action of structural maintenance of chromosome (SMC) proteins provide an additional level of regulation by organizing the genome in three dimensions (3D). Hence, the various source-sink relationships might take place in separate compartments in the nucleus.

Next, we illustrate how such source-sink relationships might provide a unified model to explain several types of epigenetic regulations, including variegation, zygotic genome activation, nucleolar dominance and the effects of mutants deficient in chromatin regulators.

## Evidence for source-sink relationships in nature

### The Y chromosome of *Drosophila* as a regulatory sink involved in selection

**Fig. 2.**
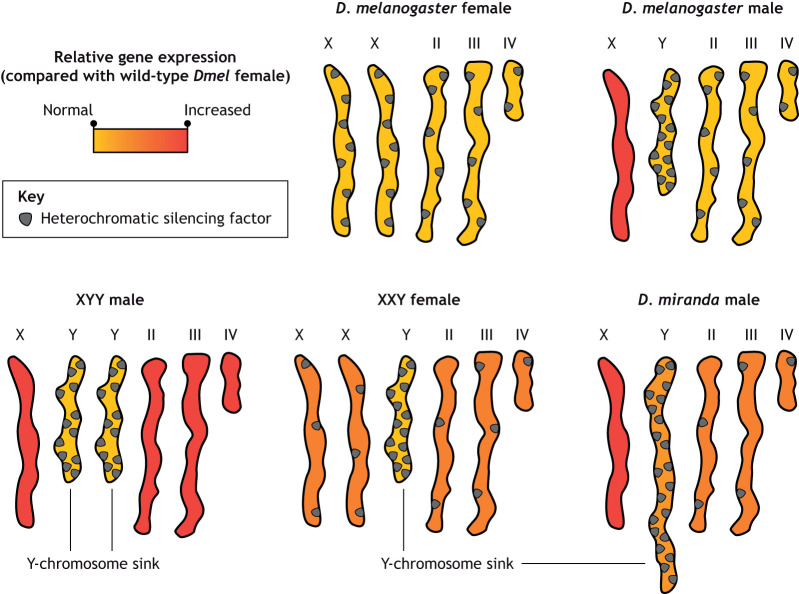
**The Y chromosome in *Drosophila* sequesters heterochromatic silencing factors away from the chromosome and autosomes.** A pictorial representation of *Drosophila* sex chromosomes across several scenarios illustrating how the size of the genomic sink increases due to the presence of the Y chromosome, leading to heterochromatic factor (gray) sequestration. Relative gene expression across chromosomes is displayed as a colorimetric heatmap when compared with wild-type *D. melanogaster* females: yellow represents normal expression and red shows overexpression.

The wealth of data on dose compensation mechanisms in *Drosophila* (Conrad and Akhtar, 2012; Samata and Akhtar, 2018) has enriched our understanding and facilitated the extension of historic observations in recent genomic analyses of sex chromosomes. The Y chromosome of *D. melanogaster* is a ∼40 Mb segment of highly repetitive heterochromatic DNA and 20 protein coding genes that accounts for ∼20% of the male haploid genome (Chang and Larracuente, 2019). It comprises satellite DNA, transposable elements (TEs) and multiple tandem repeats coding for the rRNAs. Y-linked protein-coding genes are expressed only in the male testis and are required for sperm mobility and other spermatogenetic phenotypes. The phenomenon in which quantitative effects from polymorphic Y chromosomes influence the expression of hundreds to thousands of genes located on autosomes or the X chromosome is now referred to as Y-linked regulatory variation (Lemos et al., 2008). Such a broad impact of the Y chromosome on scattered loci could be explained by dose-dependent relationships between heterochromatin, proteins and non-coding RNAs that are sensitive to mass action equilibrium (reviewed by Elgin and Reuter, 2013; Francisco and Lemos, 2014; Henikoff, 1996). A clear demonstration is provided by the effects of changing the dose of the Y chromosome on the genome-wide distribution of the major PTMs of constitutive heterochromatin (H3K9me2 and H3K9me3) in fruit flies with varying sex chromosome complements (X0, XY and XYY males; XX and XXY females). The additional Y chromosome in XYY males and XXY females acts as a sink that traps extra source factors that otherwise function to silence separate heterochromatic sites within the genome (Fig. 2). As a consequence, the depletion of heterochromatic factors by the additional Y chromosome diminishes the repression normally mediated by heterochromatin in silenced, repeat-rich regions and causes differential expression of hundreds of genes (Brown et al., 2020). Hence, an excess dose of sink Y chromosome broadly perturbs genome transcription.

In different species of *Drosophila*, changes in the Y chromosome size affect source-sink regulations. Cytogenetics and long-read sequencing indicate twofold expansion (30 to 60 Mb) in polymorphic Y chromosomes in *D. pseudoobscura*. This size difference is the result of invasion from TEs and the increased heterochromatin sink (the sum of all TEs) causes expression of Y-enriched TEs, and influences expression of dozens of autosomal and X-linked genes (Nguyen et al., 2022). In a similar manner, the recent expansion of repetitive elements on the Y chromosome of *D. miranda* corresponds with an increase in expression of TEs located within the Y chromosome, potentially indicating that *D. miranda* have not yet evolved enough silencing machinery to accommodate the recently expanded elements (Fig. 2). Accordingly, females lacking the Y chromosome are better able to silence TEs and have been shown to live longer than their male counterparts, indicating an important role of the sink Y chromosome as a regulator of fitness subjected to natural selection (Nguyen and Bachtrog, 2021).

### Changes in rDNA abundance commonly drive source-sink dynamics

**Fig. 3.**
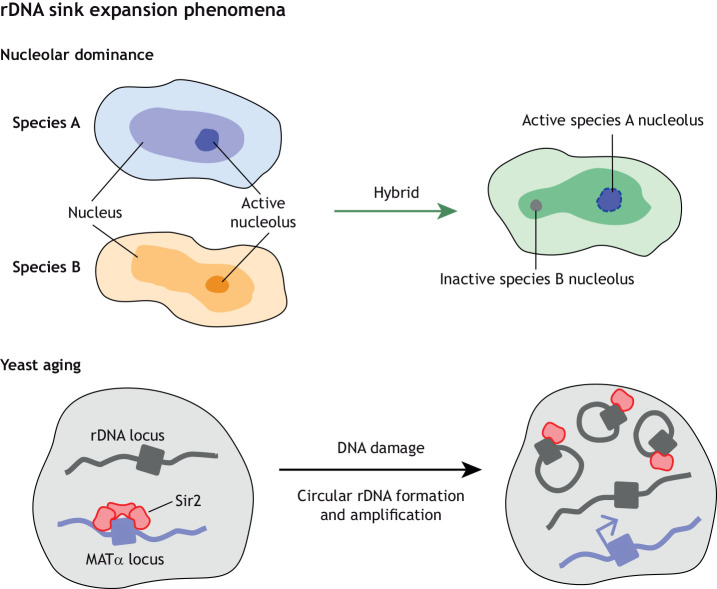
**Expansion of the rDNA sink leads to *trans* effects on transcription.** Two separate examples of rDNA expansion are provided. Nucleolar dominance (top panel) is a common phenomenon observed in many hybrid species that occurs when rDNA (located within the nucleolus) is consistently activated from only one parent species and the other parental rDNA is selectively silenced. During aging in yeast (bottom panel), DNA damage causes extra chromosomal circular rDNA species to form, and these circular DNAs sequester Sir2. This sequestration leads to de-repression of the MATα locus, causing sterility.

rRNA accounts for much of the RNA in eukaryotic cells and is encoded by hundreds to thousands of nearly identical gene copies. In diploids, individuals inherit rRNA genes from their two parents, but in hybrids, only one of the two sets of rRNA genes is actively expressed. This phenomenon is called nucleolar dominance; it was first documented in *Crepis* plant hybrids (Navashin, 1934) and later observed in many multicellular eukaryotes (Bottani et al., 2018). In *Arabidopsis*, of the two clusters of rRNA genes, only one is transcribed but there is variation in the identity of the cluster that is expressed among *A. thaliana* wild accessions. In *Arabidopsis* hybrids, nucleolar dominance is controlled by reversible, chromatin-mediated alterations in gene expression (Pontes et al., 2003; Preuss et al., 2008). rRNA gene cluster expression is controlled via complex epistatic and allelic interactions between rDNA haplotypes that apparently regulate the entire rRNA gene cluster (Rabanal et al., 2017) in a manner that likely involve balance between the dose of silencing complex activities (source) and the number of target rRNA gene clusters (sink) (Fig. 3).

In yeast, amplification of rDNA loci occurs after DNA damage and is mediated by extra chromosomal circular DNA formation, a process that emerges during yeast aging and is associated with transcriptional quiescence and cell senescence (Oberdoerffer and Sinclair, 2007). To combat circular rDNA formation, the sirtuin histone deacetylase Sir2 changes its genomic localization, exiting from the mating type loci to become bound at sites of DNA damage, including rDNA loci (Kennedy et al., 1997; Sinclair and Guarente, 1997). Sir2 relocalization relieves silencing at the mating-type loci, which results in sterility. In the context of our source-sink model, both circular rDNA formation and increased DNA damage cause the sink to expand, new sites for binding of Sir2 (the source protein) become available and secondary consequences associated with aging are the resulting outcome (Fig. 3). Interestingly, silencing of rDNA also stimulates the sequestration of Ifh1, a transcriptional co-activator of ribosomal protein genes (Albert et al., 2016), indicating that relocalization of Sir2 may secondarily lead to ribosomal protein loss and down-regulation of total mRNA translation. Remarkably, nuclear relocalization of sirtuins in response to DNA damage is conserved in mammals because two distant homologs to Sir2, SIRT1 and SIRT6, operate analogously to yeast Sir2. During the DNA damage response in mammals, SIRT1 and SIRT6 undergo relocalization and bind at double-strand breaks (Meng et al., 2020; Onn et al., 2020). Hence, the sink expansion created by DNA damage leads to removal of SIRT1 and SIRT6 from repetitive elements, which consequentially leads to de-repression of heterochromatin (Oberdoerffer et al., 2008; Simon et al., 2019). This last example outlines the interaction between different couples of source sinks (here, constitutive heterochromatin and rDNA), which opens wider possible complex modulations of source-sink modules.

### Proper source-sink relationships are crucial for developmental progression

**Fig. 4.**
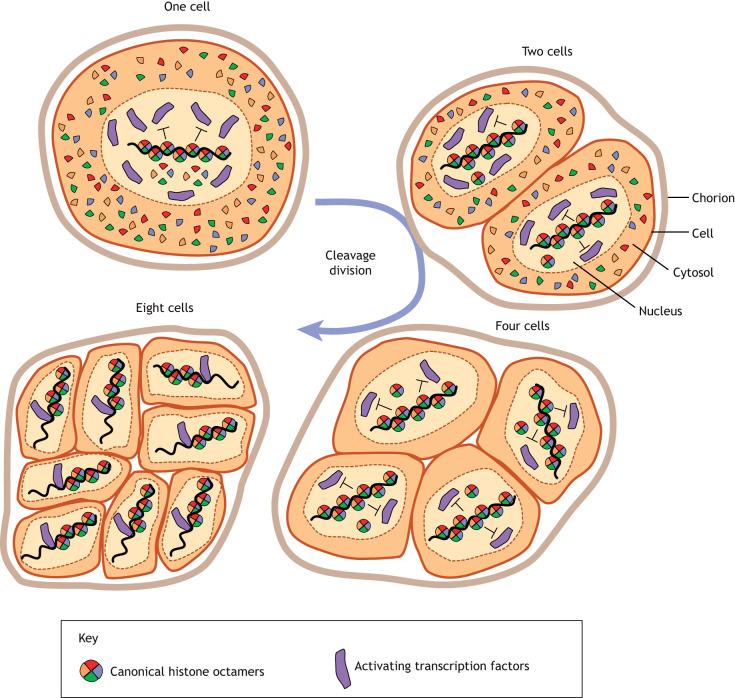
**Cleavage division during early development allows the genome sink to expand, leading to transcription factor binding and timely zygotic genome activation.** Canonical histone octamers, depicted as multicolored circles, exist in excess after fertilization (top left, one cell), but after three rounds of cell division and DNA replication (eight cells), these factors are no longer in excess. Changes in the ratio of competing sources (histones versus TF) for the same sink (DNA) allows activating transcription factors (purple) to bind, enabling transcriptional activation in the early embryo. TFs outcompete DNA binding by histones either because the TF concentration increases or because its binding affinity changes when histones are no longer in excess.

Studies of embryonic development during the maternal-to-zygotic transition provide additional illustrations supporting the source-sink model. For a range of organisms, including *Drosophila*, *Xenopus* and zebrafish, several rounds of cell division, referred to as a ‘cleavage phase’, precede the formation of stem cells during early development (Shindo et al., 2022). To ensure embryo survival, mothers must deposit enough proteins in eggs for the embryo to get through this initial period of transcriptional quiescence, and the amount of (source) proteins deposited is crucial for establishing proper developmental timing and transcriptional activation. With every cell division, more embryonic DNA is synthesized and the ratio of (source) proteins to (sink) DNA decreases (Fig. 4). Controlling how this source-sink ratio gradually decreases is crucial for the early stages of development. For example, in haploid embryos of *Xenopus*, which contain half as much DNA as diploids, the embryo reaches the appropriate ratio of protein to DNA one cell-cycle later than diploid embryos, causing zygotic genome activation to be delayed by one cell cycle (Masui and Wang, 1998). In keeping, artificially increasing the total amount of DNA present during cleavage phase causes the proper source-sink ratio to be reached earlier, leading to premature zygotic transcriptional activation (Newport and Kirschner, 1982a,b).

For organisms that undergo an extended cleavage phase of development, histone proteins are among the essential factors deposited in embryos by the mother. ‘Canonical’ histone proteins expressed from histone gene clusters in animals exist within one-cell zygotes at more than 3000-times their abundance (relative to DNA) in stem cells, and the subsequent cell divisions that occur during cleavage phase effectively dilute out these histones to appropriate levels during stem cell formation (Shindo et al., 2022) (Fig. 4). In *Xenopus*, manipulating dose of either H3/H4 histone tetramers or H2A/H2B histone dimers shifts the amount of DNA required for transcriptional activation, with excess histones causing transcription to occur at later timepoints, and decreasing histones causing transcription to occur earlier (Amodeo et al., 2015). Similar impacts have been observed in zebrafish, with the additional observation that transcriptional activators compete with excess histones for DNA-binding sites (Joseph et al., 2017), and only after histone levels are diminished (on a per-cell basis) can transcriptional activators outcompete histones for binding DNA and drive stem cell formation (Fig. 4). Interestingly, the histone variant H2A.Z seems to operate the an opposite manner, wherein excess H2A.Z installation in zebrafish embryos coincides with precocious developmental gene activation, and inhibition of genomic H2A.Z binding coincides with delayed expression (Murphy et al., 2018). Taken together, these results suggest that separate types of source proteins, ‘canonical’ histones and histones variants, may be in competition for DNA-binding sites (sinks), such that H2A.Z promotes transcriptional activation during early development, and H2A promotes silencing. Likewise, studies of *Drosophila* have found that excess amounts of ‘canonical’ histones (H3, H4, H2A and H2B) correspond with delayed zygotic genome activation (Chari et al., 2019), while the *Drosophila* homolog to H2A.Z (H2Av) accumulates on chromatin during cleavage phase (Johnson et al., 2018) and promotes transcriptional activation in embryos during maternal-to-zygotic transition (Ibarra-Morales et al., 2021). The abundance and type of ‘source’ proteins are crucial for these early stages of development, both to ensure proper developmental timing and to facilitate appropriate gene expression patterns in embryos.

## Consequences of source-sink disruption

### Mutants deficient in histone variants cause effects in *trans*

Although histone structure shows little diversity, the sequence of their intrinsically disordered loops and tails are not subject to the same constraints. This has permitted histones to diverge during eukaryotic evolution, resulting in new histone proteins referred to as histone variants (Loppin and Berger, 2020; Talbert and Henikoff, 2017, 2021). Dozens of histone variants in the H2A, H2B and H3 families have been studied not only to increase our understanding of basic chromatin biology, but also because some of these variants are associated with human diseases (Flaus et al., 2021; Oberdoerffer and Miller, 2023).

One of the most thoroughly investigated features of H2A variants is their defining sequence motifs located within their C-terminal tails. These motifs are important for the diverse mechanisms ascribed to the variants, typically via interaction with modifiers and accessory proteins, illustrated by the SQE/DΦ, the H2A.X motif (Stucki et al., 2005). Unique H2A variants associated with transcriptional repression have also evolved in animals and plants: macroH2A variants in metazoans (Guberovic et al., 2023; Sun and Bernstein, 2019) and H2A.W in vascular plants, which is distinguished by its C-terminal KSPK motif (Osakabe and Molaro, 2023; Yelagandula et al., 2014). Both the lysine-enriched linker region of macroH2A (Muthurajan et al., 2011) and the KPSK-motif of H2A.W favor heterochromatic silencing by directly changing their interaction with DNA (Lei et al., 2021; Osakabe et al., 2018; Yelagandula et al., 2014).

**Fig. 5.**
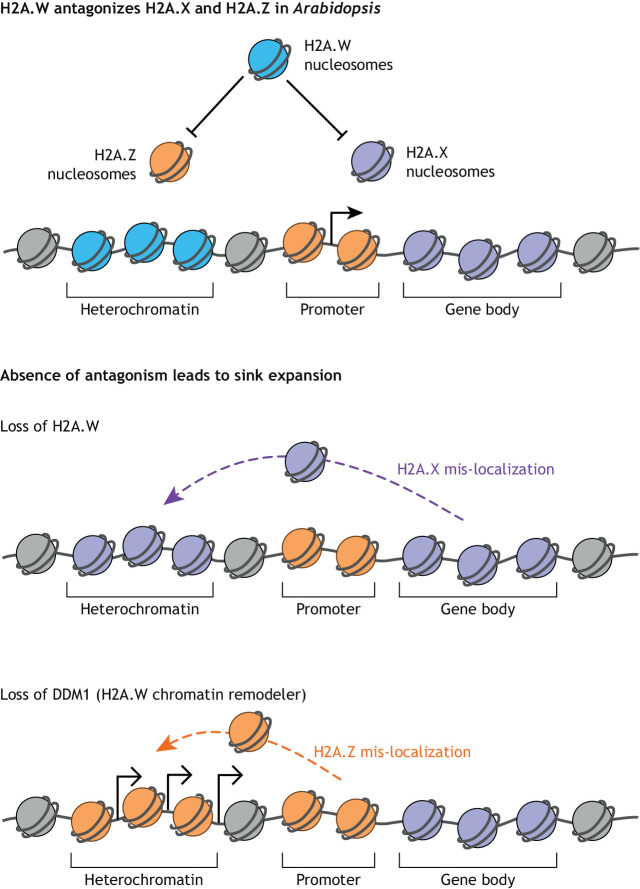
**Loss of the histone variant H2A.W at heterochromatin loci leads to sink expansion and secondary mis-localization of competing histone variants H2A.X and H2A.Z.** Nucleosomes containing the histone variants H2A.Z (orange) or H2A.X (purple) are antagonized by the histone variant H2A.W (blue). H2A.W-containing nucleosomes normally reside at heterochromatin regions (top panel), but when H2A.W is lost (middle panel), these heterochromatin loci acquire H2A.X. By contrast, when the H2A.W remodeler DDM1 is lost (bottom panel), heterochromatic regions acquire H2A.Z, which normally resides at genes repressed by H3K27me3 and promoters of active genes. In both examples, H2A.W loss leads to sink expansion and re-localization of the other histone H2A variants to heterochromatic loci.

A major theme among H2A variants is that they display highly discrete genomic localization patterns, often with context-specific functions. A clear division of histone variants into compartments has been observed in plants (Fig. 5), where each H2A variant occupies distinct territories of the genome (Osakabe et al., 2018; Yelagandula et al., 2014). In *Arabidopsis*, H2A.W is found only in constitutive heterochromatin on TEs. By contrast, H2A.Z occupies primarily protein-coding genes repressed by the Polycomb repressive complexes 1 and 2, and H2A and H2A.X occupy the bodies of expressed genes. In absence of H2A.W, this balance is upset with an invasion of heterochromatin by H2A and H2A.X, coupled with limited misexpression of hundreds of protein-coding genes and delocalization of *de novo* DNA methylation (Bourguet et al., 2021). H2A.W is deposited by the chromatin remodeler decreased in DNA methylation 1 (DDM1) to silence TEs (Osakabe et al., 2021). In contrast to mutants deficient in H2A.W, the loss of DDM1 causes a replacement of H2A.W by H2A.Z (Jamge et al., 2023). Consequently, there is massive expression of TEs and deregulation in the attribution and composition of chromatin states of constitutive and facultative heterochromatin (Jamge et al., 2023) (Fig. 5). Similarly, *Arabidopsis* mutants deficient in H2A Z cause defects in patterns of H3K27me3 and DNA methylation, affecting the expression of the genes at locations distinct from that occupied by H2A.Z the wild type (Carter et al., 2018).

The differential replacement of H2A.W by H2A.Z in *ddm1*, and of H2A.X by H2A in *h2a.w* suggest that the competition between H2A variants for occupation of heterochromatin is regulated by distinct remodelers and chaperones (Fig. 5). Similarly, the specificity of the chaperones for H3 variants is relative to their availability. For example, an excess of the centromeric H3 variants leads to H3 deposition outside centromeres by the chaperone DAXX, which is usually dedicated to the deposition of the variant H3.3 (Lacoste et al., 2014). Activity of the HIRA complex in mouse stem cells seems also to be somewhat dependent on H3.3 availability. In mouse stem cells, H3.3 is deposited at heterochromatin by the ATRX-DAXX complex, and the HIRA complex is responsible for deposition at euchromatic loci (Filipescu et al., 2014). A considerable proportion of euchromatin H3.3 resides within enhancers, and activation of these enhancers occurs with the addition of lysine 27 acetylation on H3.3 (H3.3K27ac) (Martire et al., 2019). Interestingly, levels of H3K27ac depend not only on the acetyltransferase that targets K27 and the HIRA complex that deposits H3.3, but also on the ATRX-DAXX complex, which operates exclusively over heterochromatin. Loss of the ATRX-DAXX complex coincides with significant increases in H3.3K27ac levels over euchromatic enhancers (Martire et al., 2019), suggesting that increases in HIRA activity occur when H3.3 histones not incorporated in chromatin are present in excess, and total abundance of H3.3 may govern overall enhancer activity throughout the genome. In conclusion, the competition between the histone variants and the domains where they are deposited creates a dynamic balance governed by mass action law and regulated by specific chaperones and remodelers that shape the overall chromatin landscapes.

### Mutants in silencing pathways

Mutants deprived of DNA methylation have been characterized by a general deposition of H3K7me3 over TEs in constitutive heterochromatin both in mammals and in plants. In *Arabidopsis*, the ectopic enrichment of H3K27me3 over heterochromatin in a mutant deprived of the main DNA methyltransferase 1 (MET1) is paralleled by the deprivation of H3K27me3 from its target protein-coding genes (Deleris et al., 2012). Similarly, in mutant mouse stem cells, removal of all DNA methyltransferases coincides with broad genomic increases in H3K27me3, except at the locations where H3K27me3 normally resides. These locations lose H3K27me3, perhaps at the expense of the broad accumulation occurring elsewhere (Brinkman et al., 2012). In these examples, two different source factors, DNA methyltransferases and H3K27me3 deposition enzymes, compete for the same DNA sink locations and reduction in one source factor leads to increased activity of the other. Similar antagonistic relationships have been found for the H2A.Z installation machinery and DNA methylation in several organisms, including humans (Conerly et al., 2010), zebrafish (Murphy et al., 2018) and *Arabidopsis* (Zilberman et al., 2008), suggesting that competition for sink DNA among distinct regulatory epigenetic factors (Fig. 1) may represent a general principle in biology.

Recent studies of zebrafish have found that sensitivity to environmental factors may also depend on the availability of source factors relative the sink capacity. In developing zebrafish embryos, exposure to either a common flame retardant, Tris(1,3-dichloro-2-propyl)phosphate, or a DNA methylation inhibitor, 5-aza-deoxycytidine, causes H2A.Z to accumulate specifically at transcriptionally active repetitive elements, including DNA transposons and satellite repeats (Meng et al., 2022 preprint). These changes coincide with loss of H2A.Z at promoter regions, leading to transcriptional downregulation of genes proximal to impacted loci. Remarkably, ectopically increasing the amount of available H2A.Z rescues both the molecular epigenetic impacts and the phenotypic defects in embryos, indicating that loss of H2A.Z at promoters was likely a direct result of increased H2A.Z installation at repetitive elements. In this example, separate sinks (promoters and repetitive elements) are in direct competition for the same source protein (H2A.Z) and limits on its abundance dictate how the embryo responds to environmental stressors.

Much like zebrafish embryos, failure to maintain transcriptional silencing of repetitive elements also influences the localization of specific source proteins in mammals. The silencing of endogenous TEs in mouse stem cells occurs in part through the recruitment of H3K9-methyltransferase SETDB1 and its co-repressor TRIM28 (Thompson et al., 2016). Loss of TRIM28 and/or SETDB1 not only causes re-activation of TEs, but also leads to reductions in transcriptional activation machinery over euchromatic loci, including decreased histone acetylation and loss of transcription factor binding (Asimi et al., 2022; O'Hara and Banaszynski, 2022 preprint). Most notably, binding of pluripotency factors (Oct4, Sox2 and Nanog) or RNA polymerase II is reduced over their normal genic targets in response to H3K9me3 loss, in favor of ectopic localization at de-repressed TEs. In the context of our source-sink model, this epigenetic de-repression effectively creates newly accessible DNA sinks at TEs, which become occupied by RNA polymerase and pluripotency factors (sources) at the expense of their normal target sites. Similar to the aforementioned studies of H2A.Z in zebrafish, overexpression of the pluripotency factors in mouse stem cells lacking TRIM28 rescues proper RNA polymerase II binding patterns, suggesting that activated repetitive elements are able to titrate transcription machinery away from normal sites of localization (Asimi et al., 2022). These studies indicate a broadened impact of repetitive element transcription. In addition to their mutagenic potential, these elements are seemingly able to function as a sink to sequester crucial nuclear regulatory factors, imparting dramatic influence over the rest of the genome in *trans*. Owing to the large proportion of the genome occupied by repetitive DNA elements in many organisms (>37% in mouse, >45% in humans, >50% in zebrafish and >80% in maize) (Cosby et al., 2019), it is very reasonable to suspect that their deleterious capabilities while functioning as a sink might even outweigh the negative impacts that occur when acting as a mutagen.

## Conclusions

Genetic and genomic studies altogether point to a general source-sink model to explain a large array of epigenetic regulations. Source proteins such as chromatin regulators, histones or transcription factors compete for genomic sinks within the cell. Changing availability of a source protein affects occupation of the sinks by other source proteins. In mutants that affect the wild type equilibrium, *trans* effects result from changes in occupations of sinks, consequently affecting occupation of other sinks, which accounts for the often disconcerting broad spectrum of phenotypes in mutants of chromatin regulators.

These source-sink relationships are used in wild type to regulate sequences of development. Limiting levels of source proteins appears to be an important aspect of developmental transitions because activating new loci results in ancillary silencing of loci that were previously active in progenitor cells. Hence, cells can transition to a new state or developmental lineage more easily and rapidly. If source proteins were instead in excess, then additional silencing machinery would be required to turn off previously active genes. Thus, keeping limits on sources allows the cell to effectively ‘kill two birds with one stone’. The constraints on the levels of source and the limits of the sinks set the boundaries for competition among genomic loci for source proteins, while preserving a finely tunable set of transitions from one equilibrium to another, entraining cell lineages in their respective valleys of the Waddington epigenetic landscape (Waddington, 1957). In this respect, the epigenetic source-sink relationship provides a mechanistic bridge between the molecular views and the predating Waddington's concept of epigenetics (Huang, 2022).

TEs and repetitive elements play a crucial role as their sink is modulated at the scale of the individual organisms and the evolutionary scale. At the scale of the organism, widespread de-repression of repetitive elements may allow the reservoir to re-fill, sequestering away transcription machinery while the genome undergoes epigenetic reprogramming, thus facilitating epigenetic switching on a dramatic scale. Subsequently, the reservoir can empty as repetitive loci undergo silencing and trans-factors become re-localized to genic regions. Examples of this include reprogramming events after fertilization in mammals, when de-repression of repetitive elements occurs in 2C cells, during primordial germ cell (PGC) specification in mammals when piRNAs are generated from repetitive loci, and during innate immune activation caused by ‘viral mimicry’ during cancer treatment (Chen et al., 2021; Fueyo et al., 2022; Ozata et al., 2019). This partitioning between source reservoir and receiving basin may breakdown as a consequence of aging or carcinogenesis, owing to DNA demethylation and TE de-repression, potentially causing source-factors to become inappropriately localized at TEs, limiting their use elsewhere in the genome.

Considering TEs collectively as a major epigenetic sink adds a layer of complexity to our view of their role in evolution. A sudden expansion of this sink (through chromosome rearrangements or transposon mobility) causes a dilution of the silencing sources, which are then unable to silence not only the new loci but also other TEs. This could explain bursts of transpositions causing mutations, DNA damage and malfunction of ancillary source sink. Increasing the production of source molecules, whether they are silencing or activating factors, is therefore a necessity response for survival. The wide effects of changing several source-sink relationships likely enable wide arrays of combinations of genome-wide changes submitted to selection; this might account for dramatic changes that lead to a rapid series of innovations observed in the evolutionary tree, including, notably, the evolution of sex chromosomes (Muyle et al., 2021).

Another predictable effect is the selective pressure to regulate the size of epigenetic sinks with consequences on genome size. Evolutionary studies have proposed that constraints on genome size limit the degree to which TEs are able to infiltrate genomes (Canapa et al., 2015; Kapusta et al., 2017; Schubert and Vu, 2016). One interesting possibility is that the balance between source protein levels and the size of chromatin sinks also controls genome size over the course of evolution. Based on our source-sink model, one strategy for organisms to respond to the secondary consequences of an expanding genome, faced with TE infiltration, would be to increase the abundance of source proteins. Alternatively, and perhaps more easily achieved, the organism could maintain source protein levels and instead delete unnecessary parts of the sink. With this strategy, gene expression levels could remain stable and source proteins could be re-directed to new sites of TE integration to re-establish boundaries. In support of this hypothesis, when expansion of TE-rich domains occurs, a relatively equal amount of genomic DNA is commonly lost over the same evolutionary period (Kapusta et al., 2017). However, the evolutionary pressures that establish such limits on genome sizes and/or restrict TE expansion remain unresolved (Hessen et al., 2010; Papp et al., 2011). Interestingly, large-scale genomic deletions are very common in numerous forms of cancer (Beroukhim et al., 2010), perhaps indicating that similar pressures to limit genome size may arise from disrupted source-sink dynamics during tumorigenesis. Eventually all genomes have heterochromatin, and it is possible that the sink effect of that heterochromatin is advantageous because it serves as a buffer or storage place for necessary chromatin factors. Direct manipulations of the sinks of heterochromatin via synthetic approaches might address the potential advantage or disadvantage of increased heterochromatin sink in adaptation.
